# Understanding the Self-Perceived Barriers and Enablers toward Adopting a Mediterranean Diet in Australia: An Application of the Theory of Planned Behaviour Framework

**DOI:** 10.3390/ijerph17249321

**Published:** 2020-12-13

**Authors:** Nicole Scannell, Anthony Villani, Evangeline Mantzioris, Libby Swanepoel

**Affiliations:** 1 School of Health and Behavioural Sciences, University of the Sunshine Coast, Sippy Downs, QLD 4556, Australia; nds002@student.usc.edu.au (N.S.); lswanepo@usc.edu.au (L.S.); 2 UniSA Clinical and Health Sciences, Alliance for Research in Exercise, Nutrition and Activity (ARENA), University of South Australia, Adelaide, SA 5001, Australia; Evangeline.Mantzioris@unisa.edu.au

**Keywords:** Mediterranean diet, barriers and enablers, adherence, Australia, Theory of Planned Behavior

## Abstract

The transferability of a Mediterranean diet (MedDiet) in non-Mediterranean populations is appealing. However, little is known about the perceived enablers or barriers toward adherence, particularly in Australia. This study aimed to investigate the perceived beliefs, barriers, and enablers toward adherence to a MedDiet in Australian adults. Barriers and enablers were assessed using a self-administered online questionnaire, which included questions aligned with the Theory of Planned Behaviour (TPB). The survey was completed by *n* = 606 participants. Barriers and enablers toward adherence to MedDiet were grouped under the three core constructs of the TPB: attitudes (suitability, taste, restrictive, food waste); social norms (food culture); and perceived behavioural control (PBC) (motivation, affordability, time/effort, food access, knowledge, food outlets, natural conditions, cooking skills). PBC emerged as the most prominent construct influencing intention to follow a MedDiet. Perceived health benefits (*n* = 445; 76.5%) and improved diet quality (*n* = 224; 38.5%) were identified as major advantages. In contrast, dietary adherence (*n* = 147; 39.7%) was perceived as an important disadvantage. Future MedDiet interventions, in both research and clinical settings, should consider adopting strategies aimed at improving self-efficacy to reduce self-perceived barriers and facilitate dietary adherence.

## 1. Introduction

Modifiable risk factors which promote unhealthy lifestyles, including physical inactivity and poor dietary behaviours, are among the leading contributors to non-communicable diseases (NCD) [1,2]. Accordingly, food-based dietary guidelines, including the Australian Dietary Guidelines (ADG), attempt to translate a vast evidence base regarding the relationship between foods, dietary patterns, and health-related outcomes into specific culturally acceptable dietary recommendations to reduce the overall risk of NCD. However, despite the recommendations outlined in the ADG, there is still a large portion of NCD in Australia that is attributable to lifestyle-related risk factors, including poor diet [3]. For instance, poor dietary patterns are associated with numerous NCD, including type 2 diabetes mellitus (T2DM), cancer, and cardiovascular disease (CVD) [4], all which contribute to NCD mortality rates [5]. Specifically, data from the 2015 Global Burden of Disease study revealed that one-fifth of NCD deaths were attributable to dietary risk factors, including low consumption of fruits, vegetables, nuts and seeds, and wholegrains, coupled with excessive sodium consumption [5].

The paradigm of assessing dietary patterns as opposed to single nutrients as a determinant of overall health and disease risk has been recognised for some time [6]. Over the past several decades, the Mediterranean Diet (MedDiet) has been widely investigated with respect to reductions in chronic disease risk and healthy ageing [7,8], and fits within the current model of a healthy dietary pattern for the promotion of health and disease prevention. Specifically, consistent evidence has shown that greater adherence to a MedDiet is associated with numerous health benefits, including reductions in CVD risk [9], dietary management and prevention of T2DM [10,11,12], reductions in central adiposity [10], as well as reductions in the risks associated with cancer [13], neurodegenerative conditions [14], and frailty [15]. In order to attain a greater understanding of the mechanisms associated with these proposed health benefits, adherence to the dietary pattern must be quantified. However, assessing the efficacy and adherence to a MedDiet is challenging, given the discrepancy in the interpretation of a MedDiet [16]. The traditional MedDiet is a dietary pattern consistent amongst those living in the olive-growing regions of the Mediterranean basin before the mid-1960s [17,18]. The proposed health benefits of the MedDiet and its identity are partly attributed to the consumption of traditional foods and time-honoured food-related behaviours, including harvesting and traditional culinary techniques, which are integral components of a traditional MedDiet [19,20,21,22]. In addition, there are a number of non-nutritional aspects which are suggested to indirectly contribute to a traditional MedDiet pattern, including physical activity, social, cultural, economic, and environmental features [19]. Therefore, for these reasons, a single MedDiet does not exist, as there are a number of countries represented along the Mediterranean basin [21]. Nevertheless, irrespective of its operationalisation, interpretation of a MedDiet is often characterised by a high intake of vegetables, fruits, nuts, legumes, unprocessed cereals, and daily use of extra-virgin olive oil (EVOO) incorporated into all meals; moderate consumption of fish, shellfish, fermented dairy products (cheese and yoghurt) and wine (typically during meals); low consumption of meat and meat products, processed cereals, sweets, vegetable oils, and butter [18].

It is well-recognised that changing an individual’s dietary intake is difficult and complex, as it involves making and maintaining a change in behaviour [23]. Psychological models of human behaviour, specifically dietary behaviour, can help predict this dynamic process and improve the effectiveness of an intervention [23,24,25]. Ajzen’s Theory of Planned Behaviour (TPB) identifies the biggest predictor of behaviour change is the intention to change [26]. However, intention to change is influenced by three factors: (1) attitudes, which are the personal thoughts about the change which are either favourable or non-favourable; (2) social norms (SN), which includes approval or disproval from others around them about making the change; and (3) perceived behaviour control (PBC), which refers to the perceived ease or difficulty of the behaviour change [26]. Previous evidence has shown that the combination of social and psychological factors that underpin the TPB framework strongly influence the intention to consume a healthy diet [24,27,28], as well as specific therapeutic diets [29].

Although the MedDiet is one of the most widely reported dietary patterns, the majority of evidence from robust clinical trials have previously been conducted in Mediterranean populations [20,30], with few Australian clinical trials reporting on the efficacy and adherence to a MedDiet [31]. Nevertheless, in a secondary analysis of data from the National Nutrition and Physical Activity Survey conducted in *n* = 9435 Australian adults, Aridi et al. [32] reported moderate adherence to a MedDiet when applying the Mediterranean Diet Score (MDS), developed by Trichopoulou et al. [33]. Importantly, this was one of the first Australian studies using a large, national-level cohort to show that greater adherence to a MedDiet was inversely associated with CVD risk factors [32]. Nevertheless, there is little knowledge of the perceived enablers (ease) or barriers (difficulty) that determine the behavioural intention to adhere to a MedDiet, particularly in Australia. Two previous feasibility studies delivering a MedDiet intervention targeted at Australian older adults reported that participants felt confident in their ability to maintain long-term adherence to the dietary pattern [34,35]. Irrespectively however, Davis et al. [34] reported the palatability of key foods, in particular natural Greek yoghurt, an inadequate offering of red meat, and an overall lack of variety in the diet as key barriers towards adherence. However, Zacharia et al. [35] identified the complexity of the dietary pattern, individual food preferences, and perceived cost as important barriers to adherence. Similarly, Middleton et al. [36] previously reported that middle-aged adults from the United Kingdom identified purchasing, organising, and preparing of food as key perceived barriers when attempting to adopt principles of the MedDiet. The authors also cited cultural and lifestyle barriers encountered by non-Mediterranean populations as likely obstacles toward achieving adherence to a MedDiet [36]. Moreover, perceived barriers related to knowledge, convenience, sensory appeal, and health have also been recognised as significant barriers toward adherence to a MedDiet in non-Mediterranean countries [37]. Identifying and addressing the perceived barriers toward adherence in non-Mediterranean countries, such as Australia, is an important step toward facilitating more targeted and effective implementation strategies for this population [38]. Therefore, the aim of this elicitation study is to investigate the perceived beliefs, barriers, and enablers towards adherence to a MedDiet amongst a cohort of Australian adults.

## 2. Materials and Methods

### 2.1. Participants

A cross-sectional study using a mixed methodological approach was undertaken amongst Australian males and females aged ≥18 years. Australian adults who were permanent residents of Australia and could complete an anonymous online survey in English were invited to participate. Participants were recruited via social media platforms, including Facebook, Twitter, Instagram, and LinkedIn from June 2020 to July 2020, requesting voluntary participation. Qualtrics^XM^ survey software was used to construct and distribute the survey. A link to the survey was disseminated via social media platforms where the study protocol and potential risks were clearly outlined to all interested participants. Exclusion criteria included persons <18 years of age, non-residents of Australia, or those unable to complete the survey in English.

### 2.2. Data Collection

Barriers and enablers towards following a MedDiet in Australia were assessed using a 36-item self-administered online questionnaire, designed to be completed in ~15 min. Given the lack of a validated and reliable survey instrument that can accurately assess perceived barriers and enablers towards adherence to a MedDiet, the authors developed a prototype questionnaire that was initially piloted against a separate representative sample of *n* = 15 participants for face validity. Nil changes to the readability of the questions were required following administration of the pilot questionnaire. The online questionnaire was divided into two parts. Part A consisted of open- and closed-ended questions related to participants’ demographic characteristics. An additional five open-ended questions were included to explore participants’ perceived attitudes, beliefs, barriers, and enablers towards following a MedDiet (Table 1). These five additional questions were developed to align with the TPB framework due to its effectiveness for investigating beliefs and attitudes in health-related behaviour [26]. To maximise content validity, these additional items were constructed based on accepted techniques described by Francis et al. [39]. Part B of the online questionnaire assessed participants’ adherence to a MedDiet using the previously validated 14-item Mediterranean Diet Adherence Screener (MEDAS) [40]. For the purpose of the present study, we will report on qualitative descriptive data derived from Part A of the questionnaire. Nil time restrictions were applied to complete the questionnaire, and participants were not required to answer all questions before proceeding to subsequent questions. The link to the survey was recognisable once, only to the server it was sent, thus preventing duplication when responding to the survey.

### 2.3. Data Analysis

Qualitative data were analysed using conventional content analysis [41]. Data were read and re-read for familiarisation and to determine initial codes. A recursive process was undertaken independently by two authors (NS and LS) during content analysis to maintain the rich detail of the data [42,43], and descriptions and rationale for codes were documented to confirm the reliability of the data analysis [44]. This iterative process was continued by the same two authors (NS and LS) until the research team were in agreement with the addition of a third researcher (AV) to resolve any discrepancies.

Related codes were grouped into themes; further grouping was undertaken for the themes established in the perceived barriers and enablers, using the concepts of the TPB framework. Representative quotations that illustrate the themes are presented alongside each theme and referenced with the participant number (in brackets).

Further, quantitative content analysis of response data was undertaken [45]. Identified themes were counted for frequency and percentage of responses using Microsoft Excel (2020) software. Statistical Package for the Social Sciences (SPSS) for Windows 26.0 software (IBM Corp., Armonk, NY, USA) was also used to perform analysis for the descriptive characteristics of the cohort and expressed as means ± standard deviations for continuous data, and frequencies and percentages for categorical data.

### 2.4. Ethical Approval

This study was conducted in accordance with the guidelines laid down in the Declaration of Helsinki, and all procedures involving research study participants were approved by the Research Ethics Committee (A201388), University of the Sunshine Coast, Queensland, Australia and Research Ethics Committee (203181), University of South Australia, Australia. The research involved the completion of a self-administered survey; therefore, written informed consent was implied by completion of the survey.

## 3. Results

### 3.1. Participant Characteristics

A total of *n* = 606 participants completed ≥90% of the survey and were included in the final analysis (Females, *n* = 526; Males *n* = 80). A summary of the participant demographic characteristics is presented in Table 2. More than two-thirds of participants had tertiary qualifications from university (*n* =  444; 73.3%), with one-quarter (*n* = 154; 25.4%) of participants having a nutrition- or health-related professional degree. Moreover, although participants were Australian residents, participants’ place of birth encompassed *n* = 43 different countries. Over half of all participants reported suffering various medical conditions, with depression (*n* = 72; 11.9%) and arthritis (*n* = 58; 9.6%) among the most prevalent. Lastly, over half of all respondents were classified as having middle to high income (*n =* 389; 64.2%).

### 3.2. Advantages and Disadvantages of the Mediterranean Diet

Survey responses on the advantages and disadvantages of a MedDiet were analysed for themes and presented in combination with representative quotes, frequency, and percentage of responses (Table 3). Almost all participants reported perceived advantages toward following a MedDiet (*n* = 582; 96.0%), with distinct themes identified, including health benefits, diet quality, appeal, lifestyle, affordability, and the environment. In contrast, just over 60% (*n* = 370; 61.1%) identified potential disadvantages that were grouped into the core themes of adherence, food literacy, healthfulness, convenience, taste, and culture. Identified themes are further explored below.

#### 3.2.1. Advantages

##### Health Benefits

**Figure 1:**
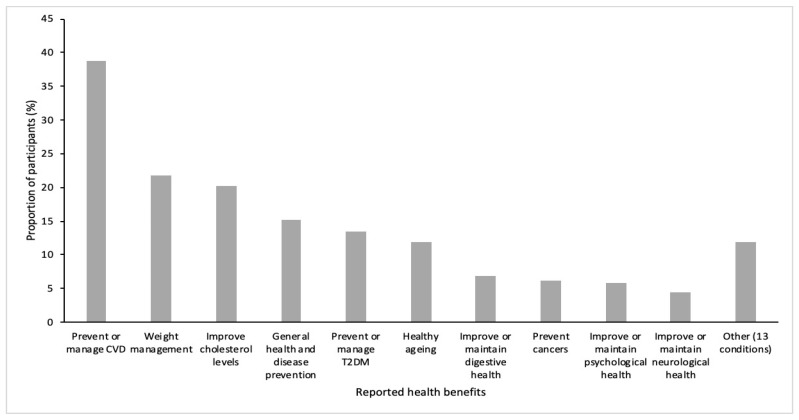
Perceived health benefits of following a Mediterranean diet, as reported by *n* = 445 survey participants. Abbreviations: CVD, Cardiovascular disease; T2DM, Type 2 diabetes mellitus.

Health benefits were identified as the most frequently reported advantage (*n* = 445; 76.5%) of following a MedDiet, with participants specifying a range of conditions for both physical and mental health. Of those who identified health benefits, management and/or prevention of cardiovascular diseases were the most frequently reported (Figure 1). Of these, a reduction in blood pressure, improving or maintaining heart health, and the prevention of stroke were the most frequently identified. In addition, many participants stated that following a MedDiet provided specific benefits for reducing high cholesterol or improving blood lipid profiles, whereas some participants also identified healthy ageing as an advantage. Moreover, participants also identified a further 13 broader health benefits, such as improved skin, greater energy, and a reduction in inflammation.

##### Diet Quality

Over one-third of participants (*n* = 224; 38.5%) believed that following a MedDiet would improve their overall diet quality, attributing this to the variety of foods and the nutrient composition of the dietary pattern. In addition, participants specifically stated that following a MedDiet encouraged a reduction in red meat consumption (*n* = 16; 2.8%), as well as the use of healthy fats or a reduction in saturated fats (*n* = 61; 10.5%), which would contribute to higher diet quality.

##### Appeal and Lifestyle

Almost one-quarter of the participants felt that the diet was appealing (*n* = 110; 18.9%) or had positive lifestyle associations (*n* = 46; 7.9%), describing it as palatable and sustainable, whilst highlighting that the potential benefits of the dietary pattern went beyond food and nutrients, but also included socialisation and enjoyment.

##### Affordability

A minority of the individuals (*n* = 15; 2.6%) felt an advantage of a MedDiet was affordability. This was attributed to buying less pre-packaged foods or prioritising the purchase of seasonal fruits and vegetables.

##### Environment

Participants that cited environmental benefits (*n* = 13; 2.2%) reported behaviours such as minimising red meat consumption and purchasing local produce as an advantage.

#### 3.2.2. Disadvantages

##### Ability to Adhere

Over one-third of participants (*n* = 147; 39.7%) stated the ability to adhere to a MedDiet was a disadvantage. Specifically, participants described a MedDiet as either too restrictive (*n* = 97; 26.2%) or overly difficult to follow (*n* = 50; 13.5) because it included unfamiliar preparation methods or was simply difficult to maintain.

##### Food Literacy

Many participants (*n* = 114; 30.8%) recognised food literacy as a disadvantage. Namely, participants identified a need for adequate knowledge of a MedDiet (*n* = 18; 4.9%), the ability to afford the perceived expense of the dietary pattern (*n* = 86; 23.2%), and a need for suitable cooking skills (*n* = 10; 2.7%).

##### “Healthfulness”

Over one-quarter of participants (*n* = 109; 29.5%) highlighted the “healthfulness” of the MedDiet as a disadvantage. Participants perceived that some of the included foods may not be healthy for those with allergies or suffering from gastrointestinal conditions. In addition, health concerns were reported for other components of the dietary pattern, including the quantity of carbohydrates, the high fat content of nuts and olive oil, the inclusion of animal products and wheat, and the overall nutritional adequacy of the dietary pattern.

##### Convenience

Convenience was identified as a disadvantage for more than one-quarter of participants (*n* = 107; 28.9%). A shortage of time secondary to family commitments and long working hours were perceived as important disadvantages, attributed to the additional effort and time required when following a MedDiet.

##### Taste

A few people (*n* = 21; 5.7%) identified taste as a disadvantage, commonly stating that the MedDiet was not palatable for themselves or other family members.

##### Culture

Lastly, some respondents (*n* = 18; 4.9%) also identified culture as a disadvantage, with some participants stating that some cultures would find it more difficult to adhere to a MedDiet due to the disparity of food flavours and social norms associated with a MedDiet.

### 3.3. Barriers and Enablers to Following a Mediterranean Diet

**Figure 2:**
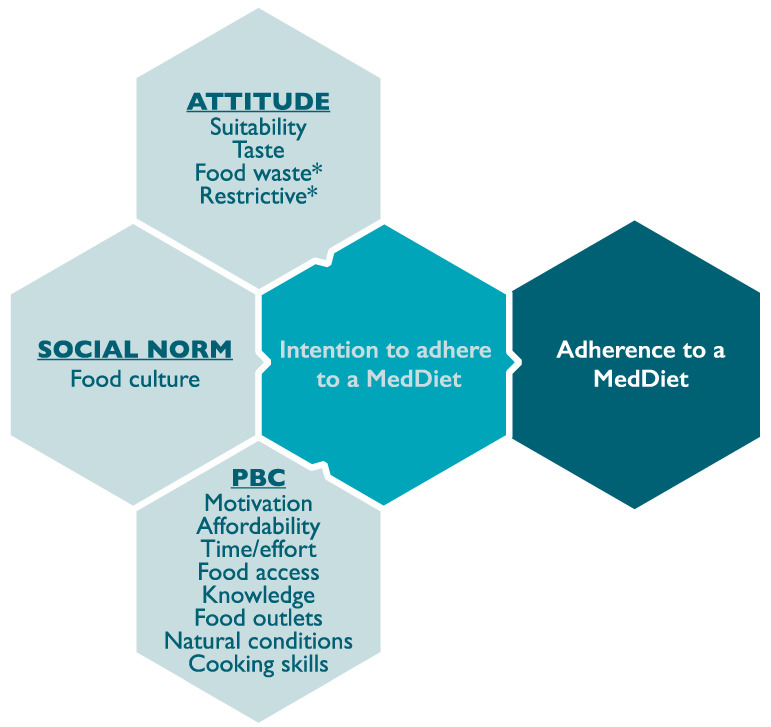
A model outlining the perceived barriers and enablers toward adherence to a MedDiet in Australian adults (model adapted from Ajzen’s Theory of Planned Behaviour [26]). Abbreviations: PBC, perceived behavioural control; * Is exclusively a perceived barrier.

Informed by the TPB framework, we propose a model which describes the perceived barriers and enablers toward following a MedDiet amongst Australian adults (Figure 2). Responses on the barriers and enablers toward following a MedDiet were analysed for common themes, which were grouped under the three core constructs of the TPB framework; attitudes, SN, and PBC. These are presented in combination with representative quotes (Table 4). Over three-quarters of all participants identified barriers (*n* = 474; 78.2%) and enablers (*n* = 507; 83.5%) toward adherence to a MedDiet. Identified themes are further explored below.

#### 3.3.1. Construct 1: Attitudes

Participants reported similar attitudes toward adherence to a MedDiet and were grouped into the themes of suitability and taste for both barriers and enablers, with the addition of restriction and food waste for reported barriers.

Suitability referred to how participants felt about the relationship between the dietary pattern and their personal dietary preferences, perceptions of healthy eating, and specific dietary requirements. A total of *n* = 48 (10.1%) participants identified suitability as a potential barrier. In contrast, *n* =14 (2.8%) participants identified it as an enabler as the dietary pattern conformed with their dietary preferences and perceptions for healthy eating. Similarly, taste and palatability were identified as a barrier for participants (*n* = 18; 3.8%) who reported disliking particular foods or cooking methods, and an enabler for participants (*n* = 10; 2%) who displayed a fondness for foods included in a Mediterranean-style dietary pattern. A small number of participants (*n* = 9; 1.9%) perceived the MedDiet to be restrictive, as they felt it omitted too many foods and displaced other culinary flavours. Lastly, an increase in waste, specifically food waste, was identified by a few participants (*n* = 4; 1%). This was attributed to the perception of the increased use of fresh produce that may go to waste if not consumed.

#### 3.3.2. Construct 2: Social Norms

Participant comments that related to social norms were all grouped into the single theme of food culture. Within this theme, participants referred to what their social groups, or the wider community thought about the MedDiet. A small proportion of participants (*n* = 7; 1.5%) expressed disapproval (e.g., family and friends) as a perceived barrier toward following a MedDiet, whereas less than 1% of participants (*n* = 3; 0.6%) identified adherence would be easier with wider acceptance among the community.

#### 3.3.3. Construct 3: Perceived Behavioural Control

Many participants described barriers and enablers toward adherence to a MedDiet in relation to PBC. Under this construct, responses were grouped into eight common themes: knowledge, food access, motivation, affordability, time/effort, cooking skills/equipment, food outlets, and environmental conditions.

Knowledge, specifically the makeup of the dietary pattern, food lists, and recipes, was identified as a potential barrier for some participants (*n* = 51; 10.8%). In contrast, a larger portion of participants (*n* = 227; 44.8%) identified that having more knowledge, particularly in relation to recipes and/or meal plans, made their acceptance of a MedDiet easier.

Many responses referred to food access, specifically in relation to acquiring fresh produce, seafood, and seasonal ingredients or proximity to local markets or supermarkets. Almost one-third of participants (*n* = 162; 32%) felt that accessibility to fishmongers and/or farmers’ markets was particularly beneficial. In contrast, one-fifth of participants (*n* = 96; 20.3%) perceived a lack of access made adherence more challenging.

Many participants conveyed motivation as a barrier (*n* = 175; 36.9%), citing the desire for other food options, including fast food or processed foods, and not wanting to change habitual dietary preferences or the food preferences of the household. Conversely, one-fifth of participants (*n* = 102; 20.1%) expressed motivation as an enabler. Specifically, participants identified that a desire to make healthy dietary changes and a congruence for this style of dietary pattern within the household made adherence less challenging.

One-quarter of respondents (*n* = 119; 25.1%) felt that affordability made it more difficult to follow a MedDiet. There was a general agreement that it was an expensive dietary pattern with frequent mention of the high cost of fruit, vegetables, and fresh seafood. In line with this common theme, almost one-fifth of participants (*n* = 92; 18.2%) reported that having greater financial resources made it easier for them to adhere to a MedDiet.

There was a preconception amongst participants that this dietary pattern would require time/effort, with particular reference to being organised when it came to food shopping, preparing, and cooking meals. Almost one-quarter of participants (*n* = 106; 22.4%) reported an increase in time/effort as a potential barrier. Accordingly, fewer participants (*n* = 67; 13.2%) identified that having time to plan and organise meals would help facilitate dietary adherence.

A few participants (*n* = 13; 2.7%) identified a lack of cooking skills and equipment as a barrier toward adherence to a MedDiet, whilst slightly more participants (*n* = 28; 5.5%) reported that confidence in the kitchen and having space to store and cook food would facilitate greater adherence.

Food outlets were identified by a small proportion of respondents (*n* = 19; 4.0%) who felt that a scarcity of eating establishments serving MedDiet-style meals and food was a barrier. A similar number of participants (*n* = 13; 2.6%) expressed that the presence of Mediterranean-style meals in restaurants and cafés would make adherence easier.

Lastly, there was consensus between the minority of participants who reported environmental conditions, such as weather, seasons, or climate as a barrier or an enabler toward adherence. Specifically, a few respondents (*n* = 5; 1.1%) felt cold conditions would make it more difficult to adhere to a MedDiet. Alternatively, a few participants (*n* = 7; 1.4%) reported that warmer weather would make adherence easier.

## 4. Discussion

Self-perceived barriers toward following a MedDiet have previously been explored [34,35,38,46,47,48]; however, to the best of our knowledge, this is the first study to comprehensively explore this relationship in Australian adults. Our results provide new insights into the factors which underpin people’s intention to follow a MedDiet. Specifically, within the context of the TPB framework, perceived behavioural control (PBC) emerged as the most predominant construct influencing intention to follow a MedDiet in our sample. In contrast, attitudes towards a MedDiet was less frequently identified, whereas social norms (SN) had little influence.

Although the transferability of a Mediterranean style diet in non-Mediterranean populations is appealing [17], embedding the principles of a MedDiet in regions with different food habits, cultural customs, and climates is potentially challenging, as this would require making and sustaining a significant change in dietary behaviour. Nevertheless, previous commentaries have eloquently articulated how various components of the MedDiet, including non-nutritional aspects, can successfully be adopted by non-Mediterranean populations [17,49], and its potential impact on public health and nutrition policies [50]. When considering the multiethnic landscape of Australia, adopting and implementing key principles of a MedDiet is possible [20], and may indeed have a substantial impact on health outcomes related to reductions in CVD and mortality risk [32,51]. Similar observations have also been reported from analysis of the European Prospective Investigation of Cancer (EPIC)-Norfolk prospective cohort study in the United Kingdom [52]. Nevertheless, the influence of PBC on dietary change is twofold, as this construct is interlinked with attitudes and SN to determine the intention to perform a behaviour, and as a precursor to actual behaviour change [24]. In the present study, the majority of barriers reported by our sample were in relation to PBC; these included motivation, affordability, time, access and knowledge. These barriers are consistent with those previously reported for both general healthy eating [53,54] and towards adopting a MedDiet in non-Mediterranean countries [36,37,46,55]. Nevertheless, complexity of the dietary pattern, individual food preferences, and perceived additional costs were previously identified as important barriers to adherence in older Australian adults [35]. These differences are likely explained by participant characteristics, with the majority of participants in the aforementioned study being retired and many reporting household incomes above the highest possible aged pension [56], suggesting time and cost are less likely to be perceived as important barriers.

Motivation to change current dietary behaviour, secondary to self-perceived difficulties in changing habitual dietary preferences, was the main perceived barrier identified by our cohort. Motivation has previously been identified as a key component in health behaviour change in theoretical models [57,58] and directly influences the intention to change behaviour [26]. In the present study, participants also identified that the food preferences of other household members easily influenced motivation. Family food preferences have consistently been identified as a key perceived barrier for adopting healthier dietary behaviours [59,60]. Akin with previous literature [60,61], we also identified that a lack of external support from others was an important barrier for facilitating dietary change. However, despite the perceived requirement of external support, the inclusion of specific strategies for improving self-efficacy remains important for facilitating dietary behaviour change. Evidence suggests that self-efficacy has a greater significance compared to social support, for facilitating and improving adherence to a MedDiet [62,63].

Non-motivational PBC factors also determine the intention to follow a MedDiet for participants in the present study. In agreement with previous literature [60], participants in our study identified a number of practical and economic barriers that hindered their dietary compliance, including time, knowledge, food access, and perceived cost implications. In a recent cross-sectional study of Italians enrolled in the Moli-sani project, a higher income was independently associated with greater adherence to a MedDiet [64]. Although we did not assess dietary adherence in the present study, our sample was generally well-educated and from a high-income bracket, yet still identified affordability and cost as an important barrier toward following a MedDiet. In a previous qualitative study amongst women in the UK [46], it was reported that the provision of a range of recipe ideas with varying time requirements coupled with cost-reducing methods would assist in changing the perception that adherence to a MedDiet is expensive. Furthermore, in a review of Australian randomized control trials using a MedDiet intervention [31], our group previously reported excellent dietary adherence, albeit with the use of a range of intensive, yet successful strategies to facilitate dietary compliance, including one-on-one dietary counselling, food provisions, written resources (recipes, daily/weekly meal plans, shopping lists, label reading information), and cooking classes. Zacharia et al. [35] also showed that acceptance of a MedDiet intervention can be improved with the provision of simplified and individualized resources (menu plans, recipes, shopping lists). Moreover, participants in this study also identified group education settings and the utilisation of mHealth strategies (website, downloadable applications, and text messaging) as preferable methods to supplement nutrition education and support behaviour change [35]. Martínez–González et al. [17] have also outlined a number of practical approaches which could be adopted by non-Mediterranean populations in order to shift a Western-style dietary pattern to a more Mediterranean-style diet. Nevertheless, in a recent national survey of Australian Dietitians, Mayr et al. [65] reported a lack of patient education resources, including practical and culturally appropriate low-cost recipe options for diverse patient groups as an important practice-related barrier for practitioners. However, implementing change to dietary and lifestyle behaviours, particularly in patients with chronic disease, is indeed complex and is likely to benefit from multi-disciplinary management in order to facilitate behavioural change [66]. In addition, providing education relating to the economic benefit of not purchasing food items that are not aligned with a MedDiet (e.g., increased fruit and vegetables may be offset by a reduction in animal products) may also be helpful [67].

Attitudes in the context of the TPB, represents an individual’s belief of the MedDiet being either positive or negative [26]. A systematic review of *n* = 19 studies analysing the relationship between TPB variables and dietary patterns showed that attitudes had the strongest association with intention to follow a dietary pattern compared with PBC and SN [24]. Contrary to this, in the present study responses describing attitudes as a determinant of behavioural intention towards adherence to a MedDiet were infrequent. Nevertheless, suitability, or the perception that the dietary pattern would not enhance the “healthfulness” of an individual’s eating behaviour, was the main attitude held by participants. This is in agreement with the barriers reported by Northern Europeans in relation to CVD risk factors, who felt that the consumption of familiar foods within a MedDiet pattern would lead to negative health outcomes, such as weight gain [48]. Therefore, promotion of the health benefits related to adherence to a MedDiet should be considered prior to adopting dietary change given that intention to adhere to a healthy dietary pattern is stronger when the dietary change is perceived to align with personal health goals [68,69].

SN were less commonly identified as influential towards the intention to follow a MedDiet by participants in this study. Participants rarely reported that the perceived external pressure from others within the community to either adopt or reject a MedDiet would influence their intention to adhere. Similarly, an examination of dietary pattern studies also showed that in the context of the TPB, SN is least associated with behaviour intention [24]. Given that food behaviour is dynamic and composed of multiple decision points [70], it is plausible that another person’s belief system imposes a diluted influence on an individual’s behaviour intention. However, the influence of SN was reported to be important in a study of overweight women participating in a diet and exercise intervention in relation to influence on meal preparation [68], suggesting further exploration is required to differentiate the influence of SN in relation to adopting a Mediterranean-style diet in Australia.

An improvement in overall diet quality was perceived as an important advantage towards following a MedDiet, with particular reference to a greater consumption of fruit and vegetables and “healthier” fats. This has previously been demonstrated in clinical trials where adherence to a MedDiet intervention was associated with an overall improvement in diet quality when compared against a habitual Australian diet [71,72,73]. Furthermore, our results also show that there is a belief that adherence to a MedDiet improves overall health and lowers the risk associated with NCDs, in particular CVD. In contrast, we found that dietary adherence was perceived as an important disadvantage. Specifically, our data suggest that cultural identity impacts food choice [74] and people are less likely to adhere to the dietary principles of a MedDiet if it is not the cultural norm [48]. Participants identified that a reduction in red meat, coupled with a greater consumption of unfamiliar foods, such as legumes, would be particularly challenging, which has previously been reported in other non-Mediterranean populations [46,48]. Inevitability, this has important implications for how a MedDiet is adopted by non-Mediterranean populations. Furthermore, this becomes multifaceted when we consider foods and their combination through cuisine, to replicate the synergistic effects of the food matrix [20]. Therefore, adapting or modernising a MedDiet to satisfy the population’s cultural views and nutritional requirements warrants attention. Previous Australian interventions exploring the efficacy of a MedDiet on cardiometabolic parameters [75,76] and cognition [77,78] have adjusted the dietary intervention to align with dietary recommendations and the culinary habits of the Australian population. Furthermore, George et al. [20] previously articulated how key dietary principles of MedDiet cuisine can be replicated across a variety of culturally specific dishes. Specifically, the authors developed a MedDiet model that conformed with principles of a traditional MedDiet and applied within a multiethnic context [20]. Additionally, the term “diet” has previously induced feelings of restriction and has been identified as a key barrier of adherence [46]. Therefore, it is conceivable that adherence to a MedDiet is viewed negatively due to the erroneous understanding of a MedDiet, where the reference towards “diet” is potentially unappealing, particularly for those who have previously attempted restrictive eating practices.

Our study is not without limitations. Specifically, we recruited a convenience sample of Australian adults who were younger, well-educated, and from a high-income bracket, which may not necessarily be generalisable to the wider population of Australian adults. Given that we used social media platforms to disseminate the questionnaire, this approach may have resulted in selection bias, given that more than 75% of the cohort recruited were <55 years old. Moreover, we did not consider living location in our analysis (urban versus rural). It is therefore possible that individuals with other educational or socioeconomic backgrounds, or indeed living location, may have identified different barriers and enablers toward adherence to a MedDiet. Specifically, there is observational evidence from children and adolescents living in Mediterranean countries suggesting that adherence to a MedDiet is higher amongst those living in small regional villages compared with larger cities [79,80]. Therefore, it is plausible that this finding may indeed be related to greater access and availability to fresh, local and seasonal foods that are consistent with a Mediterranean-style diet, such as fruit, vegetables, and fish. In the present study, we report that ~20% of participants perceived that a lack of access to farmers markets and/or fishmongers made adherence to a MedDiet more challenging. Nevertheless, our cohort was a good representation of the multi-ethnic landscape of Australia. In addition, females responded to the online questionnaire at higher rates compared with males. Therefore, it remains unclear whether barriers and enablers towards adherence to a MedDiet are indeed gendered. This is an important consideration, given that females are typically considered to be more amenable to making dietary changes than males [81,82,83,84]. Moreover, an important drawback to the qualitative nature of this study is the inability of determining whether the barriers identified are indeed “real”, or merely perceived. Further investigation into the barriers toward following a MedDiet in a heterogeneous sample of Australian adults using quantitative methodology is warranted. Future research can then be positioned to quantify key barriers and enablers for the development and validation of an appropriate tool for administration in non-Mediterranean populations.

## 5. Conclusions 

Our research contributes to the limited literature related to the perceived barriers and enablers of following a MedDiet in Australia. Using the TPB as an adopted framework, PBC, including motivation, affordability, time, access, and knowledge were identified as the most important determinants of behavioural intention toward following a MedDiet. Therefore, it would be prudent for future MedDiet interventions, in both research and clinical practice, to adopt strategies targeted at improving self-efficacy as an approach to reduce self-perceived barriers and facilitate dietary adherence amongst Australian adults. Lastly, examining the relationship between adherence to a MedDiet and a broad range of socio-demographic characteristics in non-Mediterranean populations also warrants further exploration.

## Figures and Tables

**Table 1:** Survey questions to elicit attitudes and perceived barriers and enablers toward following a Mediterranean diet.

No.	Question
1	What do you believe are the advantages of following a Mediterranean-style diet?
2	What do you believe are the disadvantages of following a Mediterranean-style diet?
3	What factors or circumstances would enable (make it easier) for you to follow a Mediterranean-style diet?
4	What factors or circumstances would make it difficult (harder) for you to follow a Mediterranean-style diet?
5	Is there anything else you associate with your own views about following a Mediterranean-style diet?

**Table 2:** Demographic characteristics of survey participants (*n* = 606).

Variable	
Gender, *n* (%)	
Males	80 (13.2)
Females	526 (86.8)
Age Category (years), *n* (%)	
18–24	57 (9.4)
25–34	149 (24.6)
35–44	163 (26.9)
45–54	93 (15.3)
55–64	84 (13.9)
65–74	46 (7.6)
Greater than 75	13 (2.1)
Not reported	1 (0.2)
BMI (kg/m^2^) *	26.1 ± 5.9
Country of birth, *n* (%)	
Australia	479 (79.0)
United Kingdom	31 (5.1)
New Zealand	19 (3.1)
South Africa	9 (1.5)
Germany	6 (1.0)
Combined 38 other countries	62 (10.3)
Highest level of education, *n* (%)	
No schooling	1 (0.2)
Secondary schooling	53 (8.7)
Trade/technical/vocational training	39 (6.4)
Diploma/Advanced diploma	69 (11.4)
Bachelor’s degree	243 (40.1)
Postgraduate degree or doctorate	201 (33.2)
Household Income AUD/year, *n* (%)	
0–24,999	30 (5.0)
25,000–74,999	135 (22.3)
75,000–124,999	178 (29.4)
125,000–199,999	151 (24.9)
200,000 or greater	60 (9.9)
Prefer not to disclose	52 (8.6)
Number of persons over 18 years in the household	2.2 ± 0.9
Reported medical conditions, *n* (%)	
No medical conditions, otherwise healthy	246 (40.6)
Depression	72 (11.9)
Arthritis	58 (9.6)
High cholesterol	47 (7.8)
High blood pressure	42 (6.9)
Type 2 diabetes mellitus	20 (3.3)
Heart disease	13 (2.1)
Osteoporosis	12 (2.0)
Neurological disease	6 (1.0)
Type 1 diabetes mellitus	6 (1.0)
Other	134 (22.1)
Not reported	83 (13.7)
Nutrition or health related qualification, *n* (%)	
Nutrition related qualification	71 (11.7)
Other health related qualification	83 (13.7)
Currently studying for nutrition related qualification	50 (8.3)
Currently studying for other health related qualification	16 (2.6)
None	372 (61.4)
Unreported	21 (3.5)

Abbreviations: BMI, body mass index. * BMI was calculated as self-reported weight (kg) divided by the square of self-reported height (m^2^).

**Table 3:** Advantages and disadvantages of a Mediterranean diet, reported by Australian adults.

	*n* (% of Total Participants)	Theme	*n* (% of Responses)		Representative Quote *
Advantages	582 (96.0)	Health benefits	445	(76.5)	“Lower cholesterol, reduce chance of developing heart disease.” (Participant 205) “Cardiovascular health, mental/brain health, decreased risk of diabetes.” (Participant 231) “Improves health of the entire body. Reduces heart disease, diabetes, cancer, depression, arthritis.” (Participant 193)
		Diet quality	224	(38.5)	“Healthier, more nutritious food.” (Participant 319) “Healthy behaviours of increased fruit and vegetable intake and reduced red meat intake.” (Participant 229) “Healthy due to good fats.” (Participant 27)
		Appeal	110	(18.9)	“It is tasty and more sustainable than other types of diets.” (Participant 581) “Delicious, full of flavours and colours.” (Participant 574) “I sincerely believe a Med diet or nutritional lifestyle plan is the ultimate for optimal health…It seems simple that “eating right” is actually not that hard.” (Participant 601)
		Lifestyle	46	(7.9)	“I believe the enjoyment of food and the social/communal aspect of eating is tantamount to the food itself.” (Participant 525) “The socialising around food, the homemade-mum food, it is related to family and social life (sic).” (Participant 565) “Family, culture, gathering over food, sharing food.” (Participant 469)
		Affordability	15	2.6	“Eating with the seasons means it is cheaper as food is in greater abundance.” (Participant 371) “It is a lot cheaper to prepare most meals from scratch.” (Participant 292) “Less financial strain by purchasing unprocessed whole foods.” (Participant 336)
		Environment	13	2.2	“Environmental sustainability (less meat consumed).” (Participant 2) “Increasing plant intake and reducing consumption of animal food may also have beneficial outcomes for the environment.” (Participant 15) “I associate a better carbon-footprint with this diet as it is focuses on local foods.” (Participant 416)
Disadvantages	370 (61.1)	Abilityto adhere	147	39.7	“We are not used to preparing food in the Mediterranean way, so it becomes daunting to attempt the diet.” (Participant 276) “Hard to stick to.” (Participant 524) “Those who aren”t used to the diet may feel it as restricting.” (Participant 477)
		Food literacy	114	30.8	“Poor food preparation knowledge and preparation skills- the diet lends itself to needing more of these skills.” (Participant 377) “Can be expensive (haloumi for example is more expensive than cheddar!).” (Participant 457) “There are many “Mediterranean” diets, so the definition is unclear. Greek Mediterranean is not the same as Italian Mediterranean.” (Participant 196)
		Healthfulness	109	29.5	“Some people have allergies to egg, dairy, wheat and nuts, some people are vegetarian, some cannot digest raw foods and legumes.” (Participant 335) “I prefer Paleo—I believe wheat is non-essential.” (Participant 563) “I cant’t follow the diet as I am vegetarian, so haven”t considered it too closely.” (Participant 468)
		Convenience	107	28.9	“Working hours, time pressures on families.” (Participant 397) “Maybe that you need to make the effort to cook your meals and think in advance.” (Participant 458) “Time to prepare food can be difficult.” (Participant 364)
		Taste	21	5.7	“I don’t actually enjoy it.” (Participant 74) “Children enjoying it.” (Participant 127) “Too oily, not enough variety.” (Participant 362)
		Culture	18	4.9	“It may be difficult for some cultures to adhere to as tastes and flavours might be much different to what they are used to.” (Participant 24) “Lack of cultural diversity.” (Participant 323) “It would likely only be applicable to those who have been raised within a Mediterranean environment and are comfortable with the types of food recommended. (i.e., a Chinese family would need a lot of social norms broken to be able to follow the Med diet).” (Participant 247)

* (Participant no.) refers to participant identification sequence in response to the questionnaire.

**Table 4:** Barriers and enablers for following a Mediterranean diet, reported by Australian adults.

		Barrier *n* = 474 (78.2%)	Enabler *n* = 507 (83.5%)	Representative Quote *	
TBP Construct	Theme	*n* (%)	*n* (%)	Barrier	Enabler
Attitude	Suitability	48 (10.1)	14 (2.8)	“Iron deficiency, reaching calcium requirements.” (Participant 591) “I’m following a plant-based wholefood diet, so find it difficult that it includes meat, fish and dairy.” (Participant 20)	“I like that it isn’t low in any of the major macronutrients, so your body doesn’t feel deprived of anything as such.” (Participant 580) “Health benefits.” (Participant 343)
	Taste	18 (3.8)	10 (2.0)	“I like plain foods…. not much oily stuff.” (Participant 540) “I am a fussy eater, for example I don’t like seafood and I think this would make it hard to follow the MS [Mediterranean style] diet.” (Participant 416)	“It is food that I enjoy eating” (Participant 296) “If I enjoyed Mediterranean style food more.” (Participant 317)
	Restrictive	9 (1.9)	-	“Very restrictive ingredients/allowed foods.” (Participant 260) “Does not encourage a range of different cultural dishes.” (Participant 582)	
	Food waste	4 (0.8)	-	“Longevity of fresh produce.” (Participant 342) “Fresh vegetables (e.g., for salad) difficult to keep fresh.” (Participant 2)	
Social Norms	Food culture	7 (1.5)	3 (0.6)	“Social networks who are against this way of eating.” (Participant 247)	“Wider acceptance and promotion throughout the community.” (Participant 425)
PBC	Motivation	175 (36.9)	102 (20.1)	“Outside influences - processed foods are too readily available.” (Participant 232)	“My friends and family following the same way of eating.” (Participant 398)
	Affordability	119 (25.1)	92 (18.2)	“[Not] being able to afford seafood.” (Participant 230)	“Cheaper options at supermarkets.” (Participant 210)
	Time/effort	106 (22.4)	67 (13.2)	“I have a newborn and I’m not sure how I would have time to strictly follow the diet.” (Participant 255)	“Less time at work.” (Participant 597)
	Food access	96 (20.3)	162 (32.0)	“Seasonal availability of produce.” (Participant 225)	“Access to fresher produce, access to affordable fresh fish.” (Participant 11)
	Knowledge	51 (10.8)	227 (44.8)	“No knowledge of what products and ingredients are best to use.” (Participant 264)	“Recipe ideas, snack ideas, lists of foods included.” (Participant 498)
	Food outlets	19 (4.0)	13 (2.6)	“Limited availability when dining out.” (Participant 328)	“Better availability in cafés/restaurants.” (Participant 405)
	Cooking skills/equipment	13 (2.7)	28 (5.5)	“If you were not a confident cook.” (Participant 321)	“Have adequate space in the kitchen for ingredients and food prep.” (Participant 3)
	Natural conditions	5 (1.1)	7 (1.4)	“Cool weather.” (Participant 170)	“Warm weather.” (Participant 323)

Abbreviations: TBP, Theory of Planned Behavior; PBC, Perceived Behavioral Control. * (Participant no.) refers to participant identification sequence in response to the questionnaire.
